# A 96-well format for a high-throughput baculovirus generation, fast titering and recombinant protein production in insect and mammalian cells

**DOI:** 10.1186/1756-0500-2-63

**Published:** 2009-04-23

**Authors:** Hanna-Riikka Kärkkäinen, Hanna P Lesch, Antti I Määttä, Pyry I Toivanen, Anssi J Mähönen, Miia M Roschier, Kari J Airenne, Olli H Laitinen, Seppo Ylä-Herttuala

**Affiliations:** 1 Department of Biotechnology and Molecular Medicine, A.I. Virtanen Institute for Molecular Sciences, Kuopio, Finland; 2 Department of Medicine, University of Kuopio, Kuopio, Finland; 3 Gene Therapy Unit, Kuopio University Hospital, Kuopio, Finland; 4 Ark Therapeutics Oyj, Kuopio, Finland

## Abstract

**Background:**

Baculovirus expression vector system (BEVS) has become a standard in recombinant protein production and virus-like particle preparation for numerous applications.

**Findings:**

We describe here protocols which adapt baculovirus generation into 96-well format.

**Conclusion:**

The established methodology allows simple baculovirus generation, fast virus titering within 18 h and efficient recombinant protein production in a high-throughput format. Furthermore, the produced baculovirus vectors are compatible with gene expression in vertebrate cells *in vitro *and *in vivo*.

## Background

Current and completed genome projects are sources of numerous novel open reading frames, which also are likely to provide new protein drug candidates or targets for medical use. This sequence information requires fluent high-throughput methods to turn into biologically relevant functional information. Over the last 20 years the safety, simplicity and capacity for the large inserts has made BEVS as a standard in recombinant protein production [1]. More recently, it has also become a popular choice for gene transfer into vertebrate cells [1-3]. Tetra-promoter vector system has previously been constructed by our group in order to achieve simple and parallel gene expression in mammalian, bacterial and insect cells with a single cloning step [4]. This universal promoter was also applied in the current study. Use of hybrid promoters active in different cell types further extends BEVS utilities. However, no reports of successful 96-well plate generation of baculoviruses are available, despite of high demand for true high-throughput screening (HTS) applications [4-6].

When expressing recombinant proteins in insect cells, or transducing vertebrate cells, amplification and titer determination of recombinant virions are important in obtaining accurate and consistent results. Often the titer is determined by detecting morphological changes in infected cells using laborious and time-consuming plaque formation and/or end-point dilution assays [7]. Several improved protocols for virus titering have been described but only a few of them allow rapid virus titering. The existing fast methods rely on troublesome protocols, reagents and facilities [8-13]. Furthermore, unlike the conventional and accepted measure of infective titer, these methods are based on viral particle or genome copy number measurements. In the current study, we describe simple 96-well protocols for the generation and fast (18 h) titering of recombinant baculoviruses.

### Construction of the baculovirus shuttle vector containing an EGFP expression cassette (F-bacmid)

**Figure 1:**
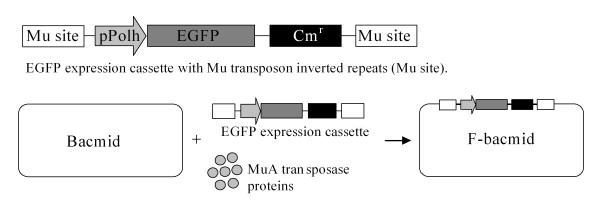
**Construction of the modified baculovirus shuttle vector, F-bacmid**. Transposition reaction was used to transfer the EGFP cassette into isolated baculovirus genome (bacmid). The MuA transposase provided the proteins needed for the integration reaction. The resultant F-bacmid was analysed for the EGFP cassette integration site. The site was found to be in the *ODV-E66 *gene.

To allow easy validation of virion production and fast titering, we first incorporated an enhanced green fluorescent proteins (EGFP) expression cassette under the control of the insect polyhedrin promoter (pPolh) into the baculovirus genome by random Mu-based transposition: The baculovirus genome (bacmid) was isolated from the DH10Bac cells (Invitrogen, Carlsbad, CA, USA) and subsequently transformed into the DH10B cells (Invitrogen). Colonies resistant for kanamycin, and sensitive to tetracycline, [14] were selected. The helper plasmid, providing Tn7-transposition proteins, was isolated similarly with the exception that the selection scheme was reversed as compared to bacmid selection. The cassette expressing EGFP was prepared by using the following procedure: pfastbac1EvoIEGFP [15] plasmid was digested with SwaI and SnaBI. The sequence containing *EGFP *gene under the polyhedrin promoter was isolated and blunted with the T4 DNA polymerase and ligated with the EcoRV-digested pEntranceposon(cam^r^) plasmid (Finnzymes, Espoo, Finland). The accomplished plasmid was cut with BglII and the fragment containing pPohl-EGFP cassette flanked by inverted terminal repeats of Mu transposon was used in transposition reaction according to manufacturer's instructions (Finnzymes). The EGFP expression cassette containing bacmids were screened for the antibiotic resistance using chloramphenicol. The resultant recombinant baculovirus genome was named as F-bacmid (Figure 1).

### Determination of the EGFP expression cassette integration site in the baculovirus genome

After introducing the EGFP expression cassette into the baculovirus genome (F-bacmid), the random integration site was determined by inverted PCR. The F-bacmid DNA was isolated, cut with BsrGI, re-ligated and linearised with FseI. Linearized DNA was amplified by PCR using 5'-ATACTCGTCGACAAGCTTCTCG-3' and 5'-GTATCAACAGGGACACCAGGAT-3' primers, which amplified the DNA outwards from the EGFP expression cassette. The amplified fragments were purified using Wizard^® ^SV Gel and PCR clean up system (Promega, Madison, WI, USA) and cloned into pGEM^®^-T Easy vectors (Promega) according to manufacturer's instructions. Clones were screened by blue-white screening and EcoRI restriction enzyme analysis. To reveal the integration site of the EGFP expression cassette, the TA-cloned inserts were sequenced with primers 5'-AATACGACTCACTATAGGG-3' and 5'-CATACGATTTAGGTGACACTATAG-3'. The obtained nucleotide sequences were fed into the NCBI-BLAST^® ^(National Centre of Biotechnology, USA- Basic Local Alignment Search Tool) search program. Sequence analysis revealed that the integration site was in the *ODV-E66 *gene. The *ODV-E66 *is a structural protein found on the envelope of occlusion derived baculovirus, but not on the budded baculovirus [16]. Thus, the integration does not disturb viral replication, but enhances safety by eliminating the formation of intact occlusion derived viruses.

### Construction of a F-bacmid containing DH10BacΔTn7 E. coli strain

To construct an *E. coli *strain that allows Tn7-mediated generation of recombinant baculoviral genomes without background [17], F-bacmid containing DH10B cells were transformed with a helper plasmid {pMON7124 [14]}. A bacterial clone was further transformed by the pBVboostΔamp plasmid [17] and selected as described earlier for the deletion of the cryptic *E.coli att*Tn7 site [17]. The strain was named as a DH10BacΔTn7EGFP.

### Generation and functionality of recombinant baculoviruses

Recombinant bacmids were prepared as described previously [4] using *E. coli *strain DH10BacΔTn7EGFP. Cells were transformed with pBVboostFG [4] donor vectors, containing either chicken avidin [18] or short form of vascular endothelial growth factor D (VEGF-DΔNΔC) [19]. These proteins represent medically and biotechnologically relevant examples of which we have years of experience. Control vectors were generated by conventional bacmids [17] and recombinant baculoviruses were produced by transfecting *Sf9 *(*Spodoptera frugiperda*, Invitrogen) insect cells as described previously [4]. Virus production in Sf9 cells was detected by fluorescence microscopy (F-bacmid viruses). Titers were determined by using end-point dilution method [7].

**Figure 2:**
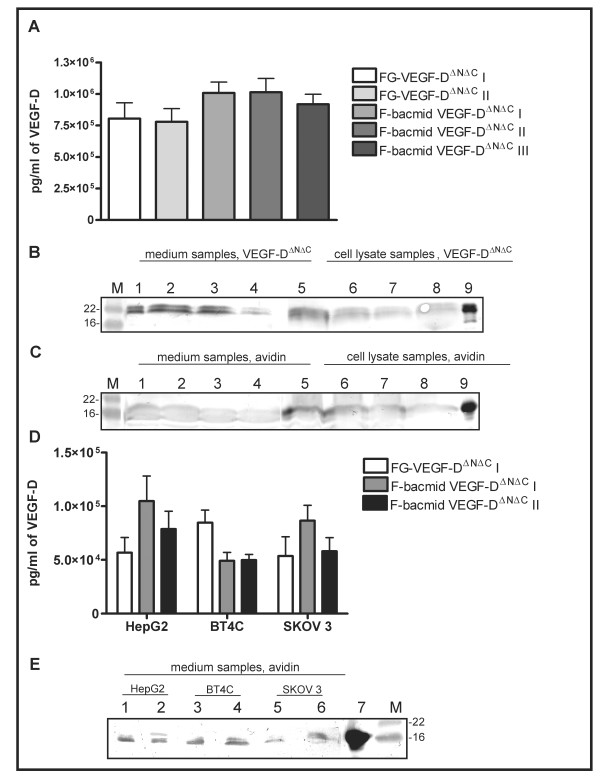
**Comparison of protein production rate of the F-bacmid and original bacmid in insect and mammalian cells**. Medium was harvested from insect and mammalian cells at 96 and 48 hours after virus addition, respectively. Equal amount of total protein was loaded into immunoblots. (A) The VEGF-D^ΔNΔC ^production in insect cells analyzed by ELISA. (B) Immunoblot of VEGF-D^ΔNΔC ^production in insect cells. Lanes 1–4 represent medium samples of FG-VEGF-D^ΔNΔC ^produced by conventional bacmid system and the three F-bacmid produced VEGF-D^ΔNΔC^. Lanes 5–8 show cell lysates from the same samples. Lane 9 is the positive control for VEGF-D. (C) Lanes 1–4 are the medium samples from two avidin controls produced by conventional bacmid system (lanes 1 and 2) compared to two F-bacmid produced avidins (lanes 3–4). Lanes 5–8 are cell lysates from the same samples. Lane 9 is positive control for avidin. (D) ELISA results for theVEGF-D^ΔNΔC ^production in mammalian cells. (E) An immunoblot analysis of avidin production in mammalian cells. Lanes 1 and 2 represent HepG2 medium samples of avidin control and F-bacmid avidin, respectively. Lanes 3–4 show the medium samples from BT4C cells, and lanes 5 and 6 from avidin produced in SKOV 3 cells. Lane 7 is the positive control for avidin. (M) Marker (SeeBlue^® ^PreStained Standard, Invitrogen).

Protein production was performed by infecting Sf9 cells in Insect X-press medium (BioWhittaker, Heidelberg, Germany) or transducing mammalian cells with produced viruses. Same viruses could be used for both cell types due to the built in universal promoter system directing transgene expression simultaneously under p10, polyhedron, T7 and chicken B-actin tetra-promoter [4]. Sf9 infection (MOI 5) was accomplished with the secondary virus preparation and allowed to continue for 4 days at 27°C, shaking at 110 rpm. Mammalian cells [Human hepatoma (HepG2), rat glioma (BT4C), and human ovarian cancer (SKOV-3)] cells were cultured in Dulbecco's Modified Eagle's Medium (DMEM, Sigma) and transduced with the unpurified secondary virus preparations (MOI 270). Fresh medium was changed after 24 hours transduction and cells were incubated for another 24 hours prior sample analysis. Both the transduced cells and medium were analyzed by immunoblotting using 1:2000 dilution of polyclonal rabbit anti-avidin [20] and 1:1000 mouse anti-hVEGF-D (R&D systems, Minneapolis, MN, USA) antibodies. The concentration of VEGF-D^ΔNΔC ^was also analyzed by ELISA (Quantikine^® ^Human VEGF-D kit). Production of avidin and VEGF-D^ΔNΔC ^by the F-bacmid viruses was comparable to the viruses produced by the original bacmid [14,17] (Figure 2). Altogether, no differences in the level of protein expression were detected in mammalian or insect cells between the bacmids. These results also indicate that the EGFP expression cassette integration into the ODV-E66 gene did not affect titer or protein production of F-bacmid derived viruses.

### Titer determination by flow cytometry

To establish a simple and fast 96-well protocol for titering of recombinant baculoviruses, the F-bacmid based EGFP expressing baculoviruses were serially diluted in duplicates into Insect X-press medium and 0.5 ml of each dilution was used to infect 0.5 ml of Sf9 cells (2 × 10^6 ^cells/ml). The infection was carried out in suspension culture that mimics well, typical large-scale virus and protein production conditions [7]. Incubation was allowed to continue for 18 or 24 hours at 27°C with shaking at 270 rpm. The samples were centrifuged for 5 minutes at 500 × g, resuspended in 2% FBS in PBS, and analyzed by flow cytometry (FACSCantoII, Becton Dickinson, Franklin Lakes, NY, USA) to reveal the percentage of the infected cells. Results were normalized according to Mulvania *et al*. [11] and virus titers were calculated (1) as an average from all dilutions which contained 1 to 10% of EGFP positive cells [21] after normalization:

(1)

**Figure 3:**
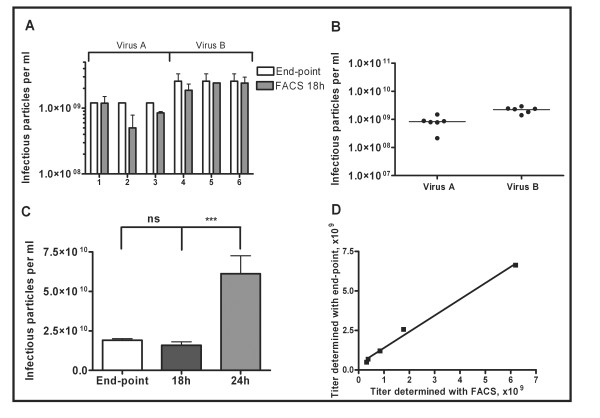
**Titer determination by flow cytometer**. (A) Titers of two virus samples (*A *and *B*) analyzed three times by the 18 h fluorescent based flow cytometric assay and end-point dilution method. (B) Consistency of the titers determined by flow cytometer after 18 hours of incubation. (C) Titer determined with flow cytometer 24 hours post infection led to overestimation of virus titer. In average titer determined 24 hours post infection were 4 times higher than the ones determined 18 hours post infection. This might be due to viral spread and secondary infections. (D) Comparison of the end-point titer and the titer determined by flow cytometer. Correlation coefficient (R = 0.9924) indicated good correlation between the two titration methods.

It was observed that 24 h incubation resulted in 4 times overestimation of the viral titer (Figure 3). This is probably due to the secondary infections due to virus production in insect cells [11]. Thus, infection time was restricted to 18 h to eliminate this possibility.

We assessed the accuracy of titers obtained with the flow cytometry by comparison to those values obtained using the end-point dilution method [7]. The titers obtained from both methods were comparable (Table 1). The end-point dilution method yielded on average 1.8 times higher titers than flow cytometric analysis. A linear regression analysis revealed an excellent correlation coefficient of 0.9924 between titers obtained using both methods (Figure 3). In addition, differences in variances were not statistically significant, further indicating reliable and constant results. As compared to the previously published titering protocols, the described FACS protocol avoids the inconvenience and expensive reagents involved in the immunostaining-based protocols [10,11,22]. To get reproducible results, only cells in exponential phase of growth and low enough passage number (below 40) was used. Good aeration was provided via shaking and by using square wells.

**Table 1:** Virus constructs and their titers obtained with end-point dilution and flow cytometer method.

	*End-point*	*SD*	*n*	*FACS*	*SD*	*n*
*F-bacmid-VEGF-D*^Δ*N*Δ*C*^*I*	6.85E+08	4.30E+08	4	3.71E+08	1.19E+08	6
*F-bacmid-VEGF-D*^Δ*N*Δ*C*^*II*	1.20E+08	0.0	2	8.44E+08	4.08E+08	6
*F-bacmid-VEGF-D*^Δ*N*Δ*C*^*III*	2.57E+09	1.06E+09	2	1.77E+09	9.55E+08	8
*F-bacmid-Avidin*	4.25E+08	2.01E+08	4	3.12E+08	8.56E+07	2
*FG-GF 1+2*	1.67E+10	3.62E+09	4	2.99E+10	2.99E+10	12
*FG-EGFP*	2.48E+10	2.40E+10	4	6.19E+09	1.66E+09	6

Titers are shown as averages of multiple analyses.

### Baculovirus generation in a 96-well plate format

Next, we tested the suitability of baculovirus production in the 96-well format. Even though there is a clear need for true high-throughput screening (HTS) methods, no reports of a successful 96-well adaptation of baculovirus generation are available [5]. Baculovirus generation was carried out by transfecting Sf9 cells in suspension using deep 96-well plates (96-well culture block, Millipore, Billericia, MA, USA) in which the shape of the well was square and the bottom was round. The transfection solution consisted of 40 μl of Insect X-press medium, 0.6 μl of cationic lipid reagent Cellfectin^® ^(Invitrogen) and 5 μl of F-bacmid DNA. The bacmid DNA was isolated with Montage BAC_96 _Miniprep Kit according to the manufacturer's instructions (Millipore). Following incubation of the transfection solution at room temperature for 30 minutes, 0.5 × 10^6 ^Sf9 insect cells in 0.5 ml of medium (Insect X-press, BioWhittaker, Heidelberg, Germany) were added. After 5 hours of incubation at 27°C with shaking at 270 rpm (Innova44 shaker, New Brunswick Scientific Edison, NJ, USA), an additional 0.5 ml of Insect X-press medium was added to the cells and the samples were further incubated.

**Figure 4:**
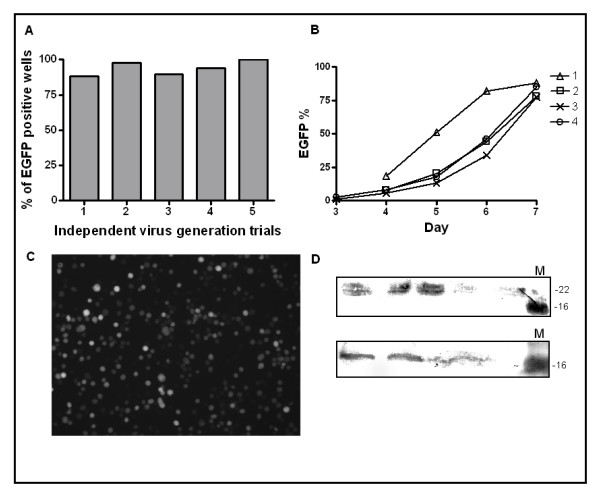
**Virus generation in 96-well format**. (A) Percentage of EGFP positive wells as a sign of virus formation in the 96-well plates in 5 independent trials. (B) Flow cytometric analysis of the EGFP percentage in independent wells from 3 to 7 days post infection. (C) A representative well showing EGFP production in F-bacmid VEGF-D^ΔNΔC ^tranfected cells 7 days post transfection (10 × magnification). (D) Protein production in 96-well plates. Sf9 cells were infected with VEGF-D^ΔNΔC ^and avidin encoding F-bacmid viruses and infection was allowed to continue for 7 days. The upper immunoblot shows VEGF-D^ΔNΔC ^production and the lower immunoblot avidin production. (M) Marker (SeeBlue^® ^PreStained Standard, Invitrogen).

Fluorescence microscopy and flow cytometry were used to detect virus production. We could reliably detect progression of virus infection in insect cells (baculovirus generation) as well as protein production. 88–100% of the 96 wells contained viruses after transfection in optimal conditions (Figure 4). The percentage of the F-bacmid (EGFP) transfected Sf9 cells increased on a daily basis between days 3 to 7 (Figure 4). At day 7, the percentage of positive cells per well was between 70 to 85% (Figure 4). Most of the previous multi-well studies show successful virus production only in the 24-well plates [5,6]. The success of virus generation in the96-well plates was obtained by extensive optimization of the infection conditions. The use of square shaped wells, vigorous shaking during incubation (ensured sufficient aeration) and suspension culturing were found to be crucial for the successful virus production.

The primary virus stocks could be used for the virus amplification and for immediate protein production, indicating high primary virus titers. Two microliters of primary virus was used to infect 1 × 10^6^Sf9 cells in one ml. The cells were incubated at 27°C, shaking at 270 rpm for 7 days. Successful protein production was detected by immunoblotting (Figure 4).

In conclusion, we present protocols which allow baculovirus generation and fast titering in a 96-well format. All virus generation steps are amenable for automation. The presented protocols are thus highly useful for widely used BEVS, as well as a basis for HTS applications and gene transfer into vertebrate cells.

## Competing interests

The authors Hanna-Riikka Kärkkäinen, Hanna P. Lesch, Pyry I. Toivanen and Anssi J Mähönen are employees of Ark Therapeutics Ltd.

## Authors' contributions

HRK contributed to study design, performed most of the experiments and drafted the manuscript. HPL contributed to study design, cloning, expression studies and manuscript drafting. AIM performed vector construction (F-bacmid) and analyzed integration site. PIT contributed to protein purification and characterization. AJM and MMR contributed to vector construction. OHL, KJA and SYH designed and supervised the experimental work, and helped on writing the manuscript. All authors read and approved the final manuscript.
